# Supplementary material for: Glycated haemoglobin and fasting plasma glucose tests in the screening of outpatients for diabetes and abnormal glucose regulation in Uganda: A diagnostic accuracy study
Source: PLoS One. 2022 Aug 4;17(8):e0272515. doi: 10.1371/journal.pone.0272515 (PMC9352087; doi:10.1371/journal.pone.0272515)
Supplement: S2 Checklist — (DOCX) [file pone.0272515.s002.docx]

**STARD for Abstracts: essential items for reporting diagnostic accuracy studies in journal or conference abstracts**

| Section | Item |
| --- | --- |
|  | Identification as a study of diagnostic accuracy using at least one measure of accuracy (such as sensitivity, specificity, predictive values, or AUC) |
| **Background and Objectives** | Study objectives |
| **Methods** | Data collection: whether this was a prospective or retrospective study |
|  | Eligibility criteria for participants and settings where the data were collected |
|  | Whether participants formed a consecutive, random, or convenience series |
|  | Description of the index test and reference standard |
| **Results** | Number of participants with and without the target condition included in the analysis |
|  | Estimates of diagnostic accuracy and their precision (such as 95% confidence intervals) |
| **Discussion** | General interpretation of the results |
|  | Implications for practice, including the intended use of the index test |
| **Registration** | Registration number and name of registry |

*Cite this as: Cohen JF, Korevaar DA, Gatsonis CA, Glasziou PP, Hooft L, Moher D, Reitsma JB, de Vet HCW, Bossuyt PM, for the STARD Group. STARD for Abstracts: Essential items for reporting diagnostic accuracy studies in journal or conference abstracts. BMJ 2017;358:j3751*
