# Supplementary material for: Glycated haemoglobin and fasting plasma glucose tests in the screening of outpatients for diabetes and abnormal glucose regulation in Uganda: A diagnostic accuracy study
Source: PLoS One. 2022 Aug 4;17(8):e0272515. doi: 10.1371/journal.pone.0272515 (PMC9352087; doi:10.1371/journal.pone.0272515)
Supplement: S1 Appendix — (DOCX) [file pone.0272515.s003.docx]

**Supporting File 1: Calculations for the Sensitivity and Specificity of the FPG and HBA_1C_ tests when used to screen for diabetes**

1. **Sensitivity and Sensitivity for FPG based on WHO and ADA cut-offs (FPG ≥ 7.0)**

| FPG_SCR_ ≥ 6.1 mmol/L | FPG_SCR_ <6.1 mmol/L |
| --- | --- |
| \|  \| OGTT ≥ 11.1 \| OGTT <11.1 \| \| --- \| --- \| --- \| \| FPG ≥ 7.0 \| 87 \| 9 \| \| FPG < 7.0 \| 23 \| 36 \| | \|  \| OGTT ≥ 11.1 \| OGTT < 11.1 \| \| --- \| --- \| --- \| \| FPG ≥ 7.0 \| 0 \| 0 \| \| FPG < 7.0 \| 3 \| 152 \| |

P(V=1|FPG =1, FPG_SCR_ ≥ 6.1mmol/L) = 1.0 P(V=1| FP =1, FPG_SCR_ <6.1 mmol/L) = 1.0

P(V=1| FPG =0, FPG_SCR_ ≥ 6.1 mmol/L) = 1.0 P(V=1| FPG =0, FPG_SCR_ < 6.1 mmol/L) = 155/1504

**Imputed tables**

| FPG_SCR_ ≥ 6.1 mmol/L | FPG_SCR_ <6.1 mmol/L |
| --- | --- |
| \|  \| OGTT ≥ 11.1 \| OGTT <11.1 \| \| --- \| --- \| --- \| \| FPG ≥ 7.0 \| 87 \| 9 \| \| FPG < 7.0 \| 23 \| 36 \| | \|  \| OGTT ≥ 11.1 \| OGTT < 11.1 \| \| --- \| --- \| --- \| \| FPG ≥ 7.0 \| 0 \| 0 \| \| FPG < 7.0 \| 29 \| 1475 \| |

**Final inverse probability weighted table**

|  | OGTT ≥ 11.1 | OGTT <11.1 |
| --- | --- | --- |
| FPG ≥ 7.0 | 87 | 9 |
| FPG < 7.0 | 52 | 1511 |

Sensitivity = $\frac{87}{139}*100$ = 62.59%

Specificity = $\frac{1511}{1520}*100$ = 99.41%

**Confidence Interval for sensitivity**

Var(logit(se)) = $\frac{1}{N}(\frac{1}{\tau\left( 1-\tau\right)}+ \frac{1-PPV}{PPVp1\tau}+\frac{NPV}{\left( 1-NPV \right)p0(1-\tau)})$

Where $\tau$=P(FPG=1) =0.0579

Since the probability of the FPG outcome in the FPG≥ 6.1 mmol/L is independent of the outcome in the FPG< 6.1, that implies that p_1_ and p_0_ is computed as below.

P_1_=P(V=1|FPG=1) = P(V=1| FPG =1, FPG≥ 6.1)* P(V=1| FPG =1, FPG< 6.1)=1.0*1.0 = 1.0

P_0_=P(V=1|FPG=0) = P(V=1| FPG =0, FPG≥ 6.1)* P(V=1| FPG =0, FPG< 6.1)=1.0*0.1031 = 0.1031

PPV=0.90625

NPV= 0.9667

=$\frac{1}{1659}(\frac{1}{0.0579\left( 1-0.0579 \right)}+ \frac{1-0.90625}{0.90625*1*0.0579}+\frac{0.9667}{\left( 1-0.9667 \right)*0.1031*(1-0.0579)}$)

= $\frac{1}{1659}(18.3326+1.7867+298.8765)$

= 0.1923

Sd(logit(se)) = 0.4385

Log($\frac{0.6259}{0.3741})$±1.96*0.4385 = (-0.3448, 1.3741)

${logit}^{-1}(-0.3448, 1.3741)$ = (0.4146, 0.7980)

The 95% CI for sensitivity

**(41.46%, 79.80%)**

**Confidence Interval for specificity**

Var(logit(sp)) = $\frac{1}{N}(\frac{1}{\tau\left( 1-\tau\right)}+ \frac{PPV}{(1-PPV)p1\tau}+\frac{1-NPV}{\left( NPV \right)p0(1-\tau)})$

Where $\tau$=P(FPG=1) =0.0579

P_1_=P(V=1|FPG=1) = P(V=1| FPG =1, FPG≥ 6.1)* P(V=1| FPG =1, FPG< 6.1)=1.0*1.0 = 1.0

P_0_=P(V=1|FPG=0) = P(V=1| FPG =0, FPG≥ 6.1)* P(V=1| FPG =0, FPG< 6.1)=1.0*0.1031 = 0.1031

PPV=0.90625

NPV= 0.9667

=$\frac{1}{1659}(\frac{1}{0.0579\left( 1-0.0579 \right)}+ \frac{0.90625}{(1-0.90625)*1*0.0579}+\frac{1-0.9667}{\left( 0.9667 \right)*0.1031*(1-0.0579)}$)

= $\frac{1}{1659}(18.3326+166.9545+0.3546)$

= 0.1119

Sd(logit(sp)) = 0.3345

Log($\frac{0.9941}{0.0059})$±1.96*0.3345 = (4.4713, 5.7825)

${logit}^{-1}(4.4713, 5.7825)$ = (0.9887, 0.9969)

The 95% CI for specificity

**(98.87%, 99.69%)**

1. **Sensitivity and Specificity for HBA1C when used to screen for diabetes based on WHO and ADA criteria**

| FPG_SCR_ ≥ 6.1 mmol/L | FPG_SCR_ <6.1 mmol/L |
| --- | --- |
| \|  \| OGTT ≥ 11.1 \| OGTT <11.1 \| \| --- \| --- \| --- \| \| HBA1c ≥ 48 \| 97 \| 3 \| \| HBA1c<48 \| 13 \| 42 \| | \|  \| OGTT ≥ 11.1 \| OGTT < 11.1 \| \| --- \| --- \| --- \| \| HBA1c≥ 48 \| 0 \| 2 \| \| HBA1c< 48 \| 3 \| 150 \| |

P(V=1|HBA1c=1, FPG_SCR_≥ 6.1) = 1.0

P(V=1|HBA1c=1, FPG_SCR_ <6.1) = 155/1504 P(V=1|HBA1c=0, FPG_SCR_ ≥ 6.1) = 1.0 P(V=1|HBA1c=0, FPG_SCR_ <6.1) = 155/1504

**Imputed tables**

| FPG_SCR_ ≥ 6.1 mmol/L | FPG_SCR_ <6.1 mmol/L |
| --- | --- |
| \|  \| OGTT ≥ 11.1 \| OGTT <11.1 \| \| --- \| --- \| --- \| \| HBA1c ≥ 48 \| 97 \| 3 \| \| HBA1c<48 \| 13 \| 42 \| | \|  \| OGTT ≥ 11.1 \| OGTT < 11.1 \| \| --- \| --- \| --- \| \| HBA1c≥ 48 \| 0 \| 19 \| \| HBA1c< 48 \| 29 \| 1455 \| |

**Final inverse probability weighted table**

|  | OGTT ≥ 11.1 | OGTT <11.1 |
| --- | --- | --- |
| HBA1c ≥ 48 | 97 | 22 |
| HBA1c<48 | 42 | 1497 |

Sensitivity = $\frac{97}{139}*100$ = 69.78%

Specificity = $\frac{1497}{1519}*100$ = 98.55%

**Confidence Interval for sensitivity for HBA1c**

Var(logit(se)) = $\frac{1}{N}(\frac{1}{\tau\left( 1-\tau\right)}+ \frac{1-PPV}{PPVp1\tau}+\frac{NPV}{\left( 1-NPV \right)p0(1-\tau)})$

Where $\tau$=P(HBA1c=1) =0.0717

Since the probability of the HBA1c outcome in the FPG≥ 6.1 mmol/L is independent of the outcome in the FPG< 6.1, that implies that p_1_ and p_0_ is computed as below.

P_1_=P(V=1|HBA1c=1) = P(V=1|HBA1c=1, FPG≥ 6.1)* P(V=1|HBA1c=1, FPG<6.1)=1.0*0.1031 = 0.1031

P_0_=P(V=1|HBA1c=0) = P(V=1|HBA1c=0, FPG≥ 6.1)* P(V=1|HBA1c=0, FPG<6.1)=1.0*0.1031 = 0.1031

PPV=0.8151

NPV= 0.9727

=$\frac{1}{1659}(\frac{1}{0.0717\left( 1-0.0717 \right)}+ \frac{1-0.8151}{0.8151*0.1031*0.0717}+\frac{0.9727}{\left( 1-0.9727 \right)*0.1031*(1-0.0717)}$)

= $\frac{1}{1659}(15.0242+30.6866+372.2796)$

= 0.2520

Sd(logit(se)) = 0.5019

Log($\frac{0.6978}{0.3022})$±1.96*0.5019 = (-0.1469, 1.821)

${logit}^{-1}(-0.1469, 1.821)$ = (0.4633, 0.8607)

The 95% CI for sensitivity

**(46.33%, 86.07%)**

**Confidence Interval for specificity**

Var(logit(sp)) = $\frac{1}{N}(\frac{1}{\tau\left( 1-\tau\right)}+ \frac{PPV}{(1-PPV)p1\tau}+\frac{1-NPV}{\left( NPV \right)p0(1-\tau)})$

Where $\tau$=P(HBA1c=1) =0.0717

P_1_=P(V=1|HBA1c=1) = P(V=1|HBA1c=1, FPG≥ 6.1)* P(V=1|HBA1c=1, FPG<6.1)=1.0*0.1031 = 0.1031

P_0_=P(V=1|HBA1c=0) = P(V=1|HBA1c=0, FPG≥ 6.1)* P(V=1|HBA1c=0, FPG<6.1)=1.0*0.1031 = 0.1031

PPV=0.8151

NPV= 0.9727

=$\frac{1}{1659}(\frac{1}{0.0717\left( 1-0.0717 \right)}+ \frac{0.8151}{(1-0.8151)*0.1031*0.0717}+\frac{1-0.9727}{\left( 0.9727 \right)*0.1031*(1-0.0717)}$)

= $\frac{1}{1659}(15.0242+596.343+0.2932)$

= 0.3687

Sd(logit(sp)) = 0.6072

Log($\frac{0.9855}{0.0145})$±1.96*0.6072 = (3.0289, 5.4091)

${logit}^{-1}(3.0289, 5.4091)$ = (0.9537, 0.9955)

The 95% CI for specificity

**(95.37%, 99.55%)**
