# Supplementary material for: Glycated haemoglobin and fasting plasma glucose tests in the screening of outpatients for diabetes and abnormal glucose regulation in Uganda: A diagnostic accuracy study
Source: PLoS One. 2022 Aug 4;17(8):e0272515. doi: 10.1371/journal.pone.0272515 (PMC9352087; doi:10.1371/journal.pone.0272515)
Supplement: S2 Appendix — (DOCX) [file pone.0272515.s004.docx]

**Supporting File 2: Calculations for the Sensitivity and Specificity of the FPG and HbA_1C_ tests when used to screen for AGR**

1. **Sensitivity and Specificity for FPG AGR based on WHO criteria**

| Verified table | Inverse probability weighted table |
| --- | --- |
| \|  \| OGTT ≥ 7.8 \| OGTT <7.8 \| \| --- \| --- \| --- \| \| FPG ≥ 6.1 \| 135 \| 20 \| \| FPG < 6.1 \| 34 \| 121 \| | \|  \| OGTT ≥ 7.8 \| OGTT < 7.8 \| \| --- \| --- \| --- \| \| FPG ≥ 6.1 \| 135 \| 20 \| \| FPG < 6.1 \| 330 \| 1174 \| |

Sensitivity = $\frac{135}{465}*100$ = 29.03%

Specificity = $\frac{1174}{1194}*100$ = 98.32%

**Confidence Interval for sensitivity**

Var(logit(se)) = $\frac{1}{N}(\frac{1}{\tau\left( 1-\tau\right)}+ \frac{1-PPV}{PPVp1\tau}+\frac{NPV}{\left( 1-NPV \right)p0(1-\tau)})$

Where $\tau$=P(FPG ≥ 6.1) = $\frac{155}{1659}$ = 0.093

P_1_=P(V=1| FPG ≥ 6.1) = 1.0

P_0_=P(V=1| FPG < 6.1) = $\frac{155}{1504}$ = 0.103

PPV= $\frac{135}{155}$ = 0.871

NPV= $\frac{1174}{1504}$ = 0.7806

=$\frac{1}{1659}(\frac{1}{0.093\left( 1-0.093 \right)}+ \frac{1-0.871}{0.871*1*0.093}+\frac{0.7806}{\left( 1-0.7806 \right)*0.103*(1-0.093)}$)

= $\frac{1}{1659}(11.8552+1.5925+38.0844)$

= 0.0311

Sd(logit(se)) = 0.1762

Log($\frac{0.2903}{0.7097})$±1.96*0.1762 = (-1.2393, -0.5486)

${logit}^{-1}(-1.2393, -0.5486)$ = (0.2246, 0.3662)

The 95% CI for sensitivity

**(22.46%, 36.62%)**

**Confidence Interval for specificity**

Var(logit(sp)) = $\frac{1}{N}(\frac{1}{\tau\left( 1-\tau\right)}+ \frac{PPV}{(1-PPV)p1\tau}+\frac{1-NPV}{\left( NPV \right)p0(1-\tau)})$

Where $\tau$=P(FPG ≥ 6.1) = $\frac{155}{1659}$ = 0.093

P_1_=P(V=1| FPG ≥ 6.1) = 1.0

P_0_=P(V=1| FPG < 6.1) = $\frac{155}{1504}$ = 0.103

PPV= $\frac{135}{155}$ = 0.871

NPV= $\frac{1174}{1504}$ = 0.7806

=$\frac{1}{1659}(\frac{1}{0.093\left( 1-0.093 \right)}+ \frac{0.871}{(1-0.871)*1*0.093}+\frac{1-0.7806}{\left( 0.7806 \right)*0.103*(1-0.093)}$)

= $\frac{1}{1659}(11.8552+72.6015+3.0086)$

= 0.0527

Sd(logit(sp)) = 0.2296

Log($\frac{0.9832}{0.0168})$±1.96*0.2296 = (3.6194, 4.5194)

${logit}^{-1}(3.6194, 4.5194)$ = (0.9739, 0.9892)

The 95% CI for specificity

**(97.39%, 98.92%)**

1. **Sensitivity and Specificity for FPG AGR based on ADA criteria**

| FPG≥ 6.1 mmol/L | FPG<6.1 mmol/L |
| --- | --- |
| \|  \| OGTT ≥ 7.8 \| OGTT < 7.8 \| \| --- \| --- \| --- \| \| FPG ≥ 5.6 \| 9 \| 6 \| \| FPG < 5.6 \| 25 \| 115 \| | \|  \| OGTT ≥ 7.8 \| OGTT < 7.8 \| \| --- \| --- \| --- \| \| FPG ≥ 5.6 \| 135 \| 20 \| \| FPG < 5.6 \| 0 \| 0 \| |

P(V=1| FPG ≥ 5.6, FPG≥ 6.1) = 1.0

P(V=1| FPG ≥ 5.6, FPG<6.1) = 155/1504 P(V=1| FPG < 5.6, FPG≥ 6.1) = 1.0 P(V=1| FPG < 5.6, FPG<6.1) = 155/1504

**Imputed tables**

| FPG≥ 6.1 mmol/L | FPG<6.1 mmol/L |
| --- | --- |
| \|  \| OGTT ≥ 7.8 \| OGTT < 7.8 \| \| --- \| --- \| --- \| \| FPG ≥ 5.6 \| 87 \| 58 \| \| FPG < 5.6 \| 243 \| 1116 \| | \|  \| OGTT ≥ 7.8 \| OGTT < 7.8 \| \| --- \| --- \| --- \| \| FPG ≥ 5.6 \| 135 \| 20 \| \| FPG < 5.6 \| 0 \| 0 \| |

**Final inverse probability weighted table**

|  | OGTT ≥ 7.8 | OGTT < 7.8 |
| --- | --- | --- |
| FPG ≥ 5.6 | 222 | 78 |
| FPG < 5.6 | 243 | 1116 |

Sensitivity = $\frac{222}{465}*100$ = 47.74%

Specificity = $\frac{1116}{1194}*100$ = 93.47%

**Confidence Interval for sensitivity**

Var(logit(se)) = $\frac{1}{N}(\frac{1}{\tau\left( 1-\tau\right)}+ \frac{1-PPV}{PPVp1\tau}+\frac{NPV}{\left( 1-NPV \right)p0(1-\tau)})$

Where $\tau$=P(FPG ≥ 5.6) = $\frac{300}{1659}$ = 0.1808

P_1_=P(V=1| FPG ≥ 5.6) = P(V=1| FPG ≥ 5.6, FPG≥ 6.1)* P(V=1| FPG ≥ 5.6, FPG<6.1)=1.0*0.1031 = 0.1031

P_0_=P(V=1| FPG < 5.6) = P(V=1| FPG < 5.6, FPG≥ 6.1)* P(V=1| FPG < 5.6, FPG<6.1)=1.0*0.1031 = 0.1031

PPV= $\frac{222}{300}$ = 0.74

NPV= $\frac{1116}{1359}$ = 0.8212

=$\frac{1}{1659}(\frac{1}{0.1808\left( 1-0.1808 \right)}+ \frac{1-0.74}{0.74*0.1031*0.1808}+\frac{0.8212}{\left( 1-0.8212 \right)*0.1031*(1-0.1808)}$)

= $\frac{1}{1659}(6.7517+18.8488+54.3792)$

= 0.0482

Sd(logit(se)) = 0.2196

Log($\frac{0.4774}{0.5226})$±1.96*0.2196 = (-0.5209, 0.33995)

${logit}^{-1}(-0.5209, 0.33995)$ = (0.3726, 0.5842)

The 95% CI for sensitivity

**(37.26%, 58.42%)**

**Confidence Interval for specificity**

Var(logit(sp)) = $\frac{1}{N}(\frac{1}{\tau\left( 1-\tau\right)}+ \frac{PPV}{(1-PPV)p1\tau}+\frac{1-NPV}{\left( NPV \right)p0(1-\tau)})$

Where $\tau$=P(FPG ≥ 5.6) = $\frac{300}{1659}$ = 0.1808

P_1_=P(V=1| FPG ≥ 5.6) = P(V=1| FPG ≥ 5.6, FPG≥ 6.1)* P(V=1| FPG ≥ 5.6, FPG<6.1)=1.0*0.1031 = 0.1031

P_0_=P(V=1| FPG < 5.6) = P(V=1| FPG < 5.6, FPG≥ 6.1)* P(V=1| FPG < 5.6, FPG<6.1)=1.0*0.1031 = 0.1031

PPV= $\frac{222}{300}$ = 0.74

NPV= $\frac{1116}{1359}$ = 0.8212

=$\frac{1}{1659}(\frac{1}{0.1808\left( 1-0.1808 \right)}+ \frac{0.74}{(1-0.74)*0.1031*0.1808}+\frac{1-0.8212}{\left( 0.8212 \right)*0.1031*(1-0.1808)}$)

= $\frac{1}{1659}(6.7517+152.6867+2.5779)$

= 0.0977

Sd(logit(sp)) = 0.3125

Log($\frac{0.9347}{0.0653})$±1.96*0.3125 = (2.0487, 3.2737)

${logit}^{-1}(2.0487, 3.2737)$ = (0.8858, 0.9635)

The 95% CI for specificity

**(88.58%, 96.35%)**

**Sensitivity and Specificity for HBA1c AGR based on ADA**

| FPG≥ 6.1 mmol/L | FPG<6.1 mmol/L |
| --- | --- |
| \|  \| OGTT ≥ 7.8 \| OGTT < 7.8 \| \| --- \| --- \| --- \| \| HbA1c≥ 39 \| 119 \| 10 \| \| HbA1c <39 \| 16 \| 10 \| | \|  \| OGTT ≥ 7.8 \| OGTT < 7.8 \| \| --- \| --- \| --- \| \| HbA1c≥ 39 \| 16 \| 34 \| \| HbA1c <39 \| 18 \| 87 \| |

P(V=1| HbA1c≥ 39, FPG≥ 6.1) = 1.0

P(V=1| HbA1c≥ 39, FPG<6.1) = 155/1504 P(V=1| HbA1c <39, FPG≥ 6.1) = 1.0 P(V=1| HbA1c <39, FPG<6.1) = 155/1504

**Imputed tables**

| FPG≥ 6.1 mmol/L | FPG<6.1 mmol/L |
| --- | --- |
| \|  \| OGTT ≥ 7.8 \| OGTT < 7.8 \| \| --- \| --- \| --- \| \| HbA1c≥ 39 \| 119 \| 10 \| \| HbA1c <39 \| 16 \| 10 \| | \|  \| OGTT ≥ 7.8 \| OGTT < 7.8 \| \| --- \| --- \| --- \| \| HbA1c≥ 39 \| 155 \| 330 \| \| HbA1c <39 \| 175 \| 844 \| |

**Final inverse probability weighted table**

|  | OGTT ≥ 7.8 | OGTT < 7.8 |
| --- | --- | --- |
| HbA1c≥ 39 | 274 | 340 |
| HbA1c <39 | 191 | 854 |

Sensitivity = $\frac{274}{465}*100$ = 58.92%

Specificity = $\frac{844}{1194}*100$ = 70.69%

**Confidence Interval for sensitivity**

Var(logit(se)) = $\frac{1}{N}(\frac{1}{\tau\left( 1-\tau\right)}+ \frac{1-PPV}{PPVp1\tau}+\frac{NPV}{\left( 1-NPV \right)p0(1-\tau)})$

Where $\tau$=P(HbA1c≥ 39) = $\frac{614}{1659}$ = 0.3701

P_1_=P(V=1| HbA1c≥ 39) = P(V=1| HbA1c≥ 39, FPG≥ 6.1)* P(V=1| HbA1c≥ 39, FPG<6.1)=1.0*0.1031 = 0.1031

P_0_=P(V=1| HbA1c <39) = P(V=1| HbA1c <39, FPG≥ 6.1)* P(V=1| HbA1c <39, FPG<6.1)=1.0*0.1031 = 0.1031

PPV= $\frac{274}{614}$ = 0.4463

NPV= $\frac{854}{1045}$ = 0.8172

=$\frac{1}{1659}(\frac{1}{0.3701\left( 1-0.3701 \right)}+ \frac{1-0.4463}{0.4463*0.1031*0.3701}+\frac{0.8172}{\left( 1-0.8172 \right)*0.1031*(1-0.3701)}$)

= $\frac{1}{1659}(4.2895+32.51396+68.83699)$

= 0.0637

Sd(logit(se)) = 0.2523

Log($\frac{0.5892}{0.4108})$±1.96*0.2523 = (-0.1338, 0.8552)

${logit}^{-1}(-0.1338, 0.8552)$ = (0.4666, 0.7017)

The 95% CI for sensitivity

**(46.66%, 70.17%)**

**Confidence Interval for specificity**

Var(logit(sp)) = $\frac{1}{N}(\frac{1}{\tau\left( 1-\tau\right)}+ \frac{PPV}{(1-PPV)p1\tau}+\frac{1-NPV}{\left( NPV \right)p0(1-\tau)})$

Where $\tau$=P(HbA1c≥ 39) = $\frac{614}{1659}$ = 0.3701

P_1_=P(V=1| HbA1c≥ 39) = P(V=1| HbA1c≥ 39, FPG≥ 6.1)* P(V=1| HbA1c≥ 39, FPG<6.1)=1.0*0.1031 = 0.1031

P_0_=P(V=1| HbA1c <39) = P(V=1| HbA1c <39, FPG≥ 6.1)* P(V=1| HbA1c <39, FPG<6.1)=1.0*0.1031 = 0.1031

PPV= $\frac{274}{614}$ = 0.4463

NPV= $\frac{854}{1045}$ = 0.8172

=$\frac{1}{1659}(\frac{1}{0.3701\left( 1-0.3701 \right)}+ \frac{0.4463}{(1-0.4463)*0.1031*0.3701}+\frac{1-0.8172}{\left( 0.8172 \right)*0.1031*(1-0.3701)}$)

= $\frac{1}{1659}(4.2895+21.1239+3.4444)$

= 0.0174

Sd(logit(sp)) = 0.1319

Log($\frac{0.7069}{0.2931})$±1.96*0.1319 = (0.6219, 1.1389)

${logit}^{-1}(0.6219, 1.1389)$ = (0.6507, 0.7575)

The 95% CI for specificity

**(65.07%, 75.75%)**
