# Supplementary material for: Glycated haemoglobin and fasting plasma glucose tests in the screening of outpatients for diabetes and abnormal glucose regulation in Uganda: A diagnostic accuracy study
Source: PLoS One. 2022 Aug 4;17(8):e0272515. doi: 10.1371/journal.pone.0272515 (PMC9352087; doi:10.1371/journal.pone.0272515)
Supplement: S3 Appendix — (DOCX) [file pone.0272515.s005.docx]

**Supporting File 3: Computation of Sensitivity and Specificity for Optimum cut-offs for Diabetes and the corresponding 95% CIs**

Based on **FPG ≥ 6.4**

FPG_SCR_ ≥ 6.1 mmol/L FPG_SCR_ <6.1 mmol/L

|  | OGTT ≥ 11.1 | OGTT <11.1 |
| --- | --- | --- |
| FPG ≥ 6.4 | 101 | 20 |
| FPG < 6.4 | 9 | 25 |

|  | OGTT ≥ 11.1 | OGTT < 11.1 |
| --- | --- | --- |
| FPG ≥ 6.4 | 0 | 0 |
| FPG < 6.4 | 3 | 152 |

P(V=1|FPG =1, FPG_SCR_ ≥ 6.1mmol/L) = 1.0 P(V=1| FP =1, FPG_SCR_ <6.1 mmol/L) = 1.0

P(V=1| FPG =0, FPG_SCR_ ≥ 6.1 mmol/L) = 1.0 P(V=1| FPG =0, FPG_SCR_ < 6.1 mmol/L) = 155/1504

**Imputed tables**

FPG_SCR_ ≥ 6.1 mmol/L FPG_SCR_ <6.1 mmol/L

|  | OGTT ≥ 11.1 | OGTT <11.1 |
| --- | --- | --- |
| FPG ≥ 6.4 | 101 | 20 |
| FPG < 6.4 | 9 | 25 |

|  | OGTT ≥ 11.1 | OGTT < 11.1 |
| --- | --- | --- |
| FPG ≥ 6.4 | 0 | 0 |
| FPG < 6.4 | 29 | 1475 |

**Final inverse probability weighted table** Sensitivity =0.7266, specificity = 0.9868

|  | OGTT ≥ 11.1 | OGTT <11.1 |
| --- | --- | --- |
| FPG ≥ 6.4 | 101 | 20 |
| FPG < 6.4 | 38 | 1500 |

**Confidence Interval for sensitivity**

Var(logit(se)) = $\frac{1}{N}(\frac{1}{\tau\left( 1-\tau\right)}+ \frac{1-PPV}{PPVp1\tau}+\frac{NPV}{\left( 1-NPV \right)p0(1-\tau)})$

Where $\tau$=P(FPG=1) =0.0729

Since the probability of the FPG outcome in the FPG≥ 6.1 mmol/L is independent of the outcome in the FPG< 6.1, that implies that p_1_ and p_0_ is computed as below.

P_1_=P(V=1|FPG=1) = P(V=1| FPG =1, FPG≥ 6.1)* P(V=1| FPG =1, FPG< 6.1)=1.0*1.0 = 1.0

P_0_=P(V=1|FPG=0) = P(V=1| FPG =0, FPG≥ 6.1)* P(V=1| FPG =0, FPG< 6.1)=1.0*0.1031 = 0.1031

PPV=0.8347

NPV= 0.9753

=$\frac{1}{1659}(\frac{1}{0.0729\left( 1-0.0729 \right)}+ \frac{1-0.8347}{0.8347*1*0.0729}+\frac{0.9753}{\left( 1-0.9753 \right)*0.1031*(1-0.0729)}$)

= $\frac{1}{1659}(14.7961+2.7165+413.1008)$

= 0.2596

Sd(logit(se)) = 0.5095

Log($\frac{0.7266}{0.2734})$±1.96*0.5095 = (-0.0212, 1.9761)

${logit}^{-1}(-0.0212, 1.9761)$ = (0.4947, 0.8783)

The 95% CI for sensitivity

**(49.47%, 87.83%)**

**Confidence Interval for specificity**

Var(logit(sp)) = $\frac{1}{N}(\frac{1}{\tau\left( 1-\tau\right)}+ \frac{PPV}{(1-PPV)p1\tau}+\frac{1-NPV}{\left( NPV \right)p0(1-\tau)})$

Where $\tau$=P(FPG=1) =0.0729

P_1_=P(V=1|FPG=1) = P(V=1| FPG =1, FPG≥ 6.1)* P(V=1| FPG =1, FPG< 6.1)=1.0*1.0 = 1.0

P_0_=P(V=1|FPG=0) = P(V=1| FPG =0, FPG≥ 6.1)* P(V=1| FPG =0, FPG< 6.1)=1.0*0.1031 = 0.1031

PPV=0.8347

NPV= 0.9753

=$\frac{1}{1659}(\frac{1}{0.0729\left( 1-0.0729 \right)}+ \frac{0.8347}{(1-0.8347)*1*0.0729}+\frac{1-0.9753}{\left( 0.9753 \right)*0.1031*(1-0.0729)}$)

= $\frac{1}{1659}(14.7961+69.2676+0.2650)$

= 0.0508

Sd(logit(sp)) = 0.2255

Log ($\frac{0.9868}{0.0132})$±1.96*0.2255 = 4.31425±1.96*0.2255 = (3.8723, 4.7562)

${logit}^{-1}(3.8723, 4.7562)$ = (0.9796, 0.9915)

The 95% CI for specificity

**(****97.96%, 99.15%)**

Based on **HBA1c≥ 45**

FPG_SCR_ ≥ 6.1 mmol/L FPG_SCR_ <6.1 mmol/L

|  | OGTT ≥ 11.1 | OGTT <11.1 |
| --- | --- | --- |
| HBA1c ≥ 45 | 99 | 6 |
| HBA1c < 45 | 11 | 39 |

|  | OGTT ≥ 11.1 | OGTT < 11.1 |
| --- | --- | --- |
| HBA1c ≥ 45 | 0 | 7 |
| HBA1c < 45 | 3 | 145 |

**Imputed tables**

FPG_SCR_ ≥ 6.1 mmol/L FPG_SCR_ <6.1 mmol/L

|  | OGTT ≥ 11.1 | OGTT <11.1 |
| --- | --- | --- |
| HBA1c ≥ 45 | 99 | 6 |
| HBA1c < 45 | 11 | 39 |

|  | OGTT ≥ 11.1 | OGTT < 11.1 |
| --- | --- | --- |
| HBA1c ≥ 45 | 0 | 68 |
| HBA1c < 45 | 29 | 1407 |

**Final inverse probability weighted table** Sensitivity =0.7122, specificity = 0.9513

|  | OGTT ≥ 11.1 | OGTT <11.1 |
| --- | --- | --- |
| HBA1c ≥ 45 | 99 | 74 |
| HBA1c < 45 | 40 | 1446 |

**Confidence Interval for sensitivity for HBA1c**

Var(logit(se)) = $\frac{1}{N}(\frac{1}{\tau\left( 1-\tau\right)}+ \frac{1-PPV}{PPVp1\tau}+\frac{NPV}{\left( 1-NPV \right)p0(1-\tau)})$

Where $\tau$=P(HBA1c=1) =0.1043

Since the probability of the HBA1c outcome in the FPG≥ 6.1 mmol/L is independent of the outcome in the FPG< 6.1, that implies that p_1_ and p_0_ is computed as below.

P_1_=P(V=1|HBA1c=1) = P(V=1|HBA1c=1, FPG≥ 6.1)* P(V=1|HBA1c=1, FPG<6.1)=1.0*0.1031 = 0.1031

P_0_=P(V=1|HBA1c=0) = P(V=1|HBA1c=0, FPG≥ 6.1)* P(V=1|HBA1c=0, FPG<6.1)=1.0*0.1031 = 0.1031

PPV=0.5723

NPV= 0.9731

=$\frac{1}{1659}(\frac{1}{0.1043\left( 1-0.1043 \right)}+ \frac{1-0.5723}{0.5723*0.1031*0.1043}+\frac{0.9731}{\left( 1-0.9731 \right)*0.1031*(1-0.1043)}$)

= $\frac{1}{1659}(10.7042+69.498+391.7274)$

= 0.2845

Sd(logit(se)) = 0.5334

Log($\frac{0.7122}{0.2878})$±1.96*0.5334 = (-0.1394, 1.9516)

${logit}^{-1}(-0.1394, 1.9516)$ = (0.4652, 0.8756)

The 95% CI for sensitivity

**(46.52%, 87.56%)**

**Confidence Interval for specificity**

Var(logit(sp)) = $\frac{1}{N}(\frac{1}{\tau\left( 1-\tau\right)}+ \frac{PPV}{(1-PPV)p1\tau}+\frac{1-NPV}{\left( NPV \right)p0(1-\tau)})$

Where $\tau$=P(HBA1c=1) =0.1043

P_1_=P(V=1|HBA1c=1) = P(V=1|HBA1c=1, FPG≥ 6.1)* P(V=1|HBA1c=1, FPG<6.1)=1.0*0.1031 = 0.1031

P_0_=P(V=1|HBA1c=0) = P(V=1|HBA1c=0, FPG≥ 6.1)* P(V=1|HBA1c=0, FPG<6.1)=1.0*0.1031 = 0.1031

PPV=0.5723

NPV= 0.9731

=$\frac{1}{1659}(\frac{1}{0.1043\left( 1-0.1043 \right)}+ \frac{0.5723}{(1-0.5723)*0.1031*0.1043}+\frac{1-0.9731}{\left( 0.9731 \right)*0.1031*(1-0.1043)}$)

= $\frac{1}{1659}(10.7042+124.4347+0.2993)$

= 0.0816

Sd(logit(sp)) = 0.2857

Log($\frac{0.9513}{0.0487})$±1.96*0.2857= (2.4122, 3.5321)

${logit}^{-1}(2.4122, 3.5321)$ = (0.9178, 0.9716)

The 95% CI for specificity

**(91.78%, 97.16%)**

**Computation of Sensitivity and Specificity for New cutoffs for AGR and 95% CI**

Based on **FPG ≥ 5.5**

FPG_SCR_ ≥ 6.1 mmol/L FPG_SCR_ <6.1 mmol/L

|  | OGTT ≥ 7.8 | OGTT <7.8 |
| --- | --- | --- |
| FPG ≥ 5.5 | 135 | 20 |
| FPG < 5.5 | 0 | 0 |

|  | OGTT ≥ 7.8 | OGTT <7.8 |
| --- | --- | --- |
| FPG ≥ 5.5 | 11 | 7 |
| FPG < 5.5 | 22 | 115 |

P(V=1|FPG =1, FPG_SCR_ ≥ 6.1mmol/L) = 1.0 P(V=1| FP =1, FPG_SCR_ <6.1 mmol/L) = 1.0

P(V=1| FPG =0, FPG_SCR_ ≥ 6.1 mmol/L) = 1.0 P(V=1| FPG =0, FPG_SCR_ < 6.1 mmol/L) = 155/1504

**Imputed tables**

FPG_SCR_ ≥ 6.1 mmol/L FPG_SCR_ <6.1 mmol/L

|  | OGTT ≥ 7.8 | OGTT <7.8 |
| --- | --- | --- |
| FPG ≥ 5.5 | 135 | 20 |
| FPG < 5.5 | 0 | 0 |

|  | OGTT ≥ 7.8 | OGTT <7.8 |
| --- | --- | --- |
| FPG ≥ 5.5 | 107 | 68 |
| FPG < 5.5 | 213 | 1116 |

**Final inverse probability weighted table** Sensitivity =0.5319, specificity = 0.9269

|  | OGTT ≥ 7.8 | OGTT <7.8 |
| --- | --- | --- |
| FPG ≥ 5.5 | 242 | 88 |
| FPG < 5.5 | 213 | 1116 |

**Confidence Interval for sensitivity**

Var(logit(se)) = $\frac{1}{N}(\frac{1}{\tau\left( 1-\tau\right)}+ \frac{1-PPV}{PPVp1\tau}+\frac{NPV}{\left( 1-NPV \right)p0(1-\tau)})$

Where $\tau$=P(FPG=1) =0.1989

Since the probability of the FPG outcome in the FPG≥ 6.1 mmol/L is independent of the outcome in the FPG< 6.1, that implies that p_1_ and p_0_ is computed as below.

P_1_=P(V=1|FPG=1) = P(V=1| FPG =1, FPG≥ 6.1)* P(V=1| FPG =1, FPG< 6.1)=1.0*1.0 = 1.0

P_0_=P(V=1|FPG=0) = P(V=1| FPG =0, FPG≥ 6.1)* P(V=1| FPG =0, FPG< 6.1)=1.0*0.1031 = 0.1031

PPV=0.7333

NPV= 0.8397

=$\frac{1}{1659}(\frac{1}{0.1989\left( 1-0.1989 \right)}+ \frac{1-0.7333}{0.7333*1*0.1989}+\frac{0.8397}{\left( 1-0.8397 \right)*0.1031*(1-0.1989)}$)

= $\frac{1}{1659}(6.2759+1.8285+63.4228)$

= 0.0431

Sd(logit(se)) = 0.2076

Log($\frac{0.5319}{0.4681})$±1.96*0.2076 = (-0.2791, 0.5347)

${logit}^{-1}(-0.2791, 0.5347)$ = (0.4307, 0.6306)

The 95% CI for sensitivity

**(43.07%, 63.06%)**

**Confidence Interval for specificity**

Var(logit(sp)) = $\frac{1}{N}(\frac{1}{\tau\left( 1-\tau\right)}+ \frac{PPV}{(1-PPV)p1\tau}+\frac{1-NPV}{\left( NPV \right)p0(1-\tau)})$

Where $\tau$=P(FPG=1) =0.1989

P_1_=P(V=1|FPG=1) = P(V=1| FPG =1, FPG≥ 6.1)* P(V=1| FPG =1, FPG< 6.1)=1.0*1.0 = 1.0

P_0_=P(V=1|FPG=0) = P(V=1| FPG =0, FPG≥ 6.1)* P(V=1| FPG =0, FPG< 6.1)=1.0*0.1031 = 0.1031

PPV=0.7333

NPV= 0.8397

=$\frac{1}{1659}(\frac{1}{0.1989\left( 1-0.1989 \right)}+ \frac{0.7333}{(1-0.7333)*1*0.1989}+\frac{1-0.8397}{\left( 0.8397 \right)*0.1031*(1-0.1989)}$)

= $\frac{1}{1659}(6.2759+13.8237+2.3113)$

= 0.0135

Sd(logit(sp)) = 0.1162

Log($\frac{0.9269}{0.0731})$±1.96*0.1162 = (2.3123, 2.7678)

${logit}^{-1}(2.3123, 2.7678)$ = (0.9099, 0.9409)

The 95% CI for specificity

**(90.99%, 94.09%)**

Based on **HBA1c≥ 42**

FPG_SCR_ ≥ 6.1 mmol/L FPG_SCR_ <6.1 mmol/L

|  | OGTT ≥ 7.8 | OGTT <7.8 |
| --- | --- | --- |
| HBA1c ≥ 42 | 115 | 5 |
| HBA1c < 42 | 20 | 15 |

|  | OGTT ≥ 7.8 | OGTT <7.8 |
| --- | --- | --- |
| HBA1c ≥ 42 | 8 | 13 |
| HBA1c < 42 | 25 | 109 |

**Imputed tables**

FPG_SCR_ ≥ 6.1 mmol/L FPG_SCR_ <6.1 mmol/L

|  | OGTT ≥ 7.8 | OGTT <7.8 |
| --- | --- | --- |
| HBA1c ≥ 42 | 115 | 5 |
| HBA1c < 42 | 20 | 15 |

|  | OGTT ≥ 7.8 | OGTT <7.8 |
| --- | --- | --- |
| HBA1c ≥ 42 | 78 | 126 |
| HBA1c < 42 | 243 | 1058 |

|  | OGTT ≥ 7.8 | OGTT <7.8 |
| --- | --- | --- |
| HBA1c ≥ 42 | 193 | 131 |
| HBA1c < 42 | 263 | 1073 |

**Final inverse probability weighted table**

Sensitivity =0.4232, specificity = 0.8912

**Confidence Interval for sensitivity for HBA1c**

Var(logit(se)) = $\frac{1}{N}(\frac{1}{\tau\left( 1-\tau\right)}+ \frac{1-PPV}{PPVp1\tau}+\frac{NPV}{\left( 1-NPV \right)p0(1-\tau)})$

Where $\tau$=P(HBA1c=1) =0.1953

Since the probability of the HBA1c outcome in the FPG≥ 6.1 mmol/L is independent of the outcome in the FPG< 6.1, that implies that p_1_ and p_0_ is computed as below.

P_1_=P(V=1|HBA1c=1) = P(V=1|HBA1c=1, FPG≥ 6.1)* P(V=1|HBA1c=1, FPG<6.1)=1.0*0.1031 = 0.1031

P_0_=P(V=1|HBA1c=0) = P(V=1|HBA1c=0, FPG≥ 6.1)* P(V=1|HBA1c=0, FPG<6.1)=1.0*0.1031 = 0.1031

PPV=0.5957

NPV= 0.8031

=$\frac{1}{1659}(\frac{1}{0.1953\left( 1-0.1953 \right)}+ \frac{1-0.5957}{0.5957*0.1031*0.1953}+\frac{0.8031}{\left( 1-0.8031 \right)*0.1031*(1-0.1953)}$)

= $\frac{1}{1659}(6.3630+33.7066+49.162)$

= 0.0538

Sd(logit(se)) = 0.2319

Log($\frac{0.4232}{0.5768})$±1.96*0.2319 = (-0.7642, 0.1449)

${logit}^{-1}(-0.7642, 0.1449)$ = (0.3177, 0.5362)

The 95% CI for sensitivity

**(31.77%, 53.62%)**

**Confidence Interval for specificity**

Var(logit(sp)) = $\frac{1}{N}(\frac{1}{\tau\left( 1-\tau\right)}+ \frac{PPV}{(1-PPV)p1\tau}+\frac{1-NPV}{\left( NPV \right)p0(1-\tau)})$

Where $\tau$=P(HBA1c=1) =0.1953

P_1_=P(V=1|HBA1c=1) = P(V=1|HBA1c=1, FPG≥ 6.1)* P(V=1|HBA1c=1, FPG<6.1)=1.0*0.1031 = 0.1031

P_0_=P(V=1|HBA1c=0) = P(V=1|HBA1c=0, FPG≥ 6.1)* P(V=1|HBA1c=0, FPG<6.1)=1.0*0.1031 = 0.1031

PPV=0.5957

NPV= 0.8031

=$\frac{1}{1659}(\frac{1}{0.1953\left( 1-0.1953 \right)}+ \frac{0.5957}{(1-0.5957)*0.1031*0.1953}+\frac{1-0.8031}{\left( 0.8031 \right)*0.1031*(1-0.1953)}$)

= $\frac{1}{1659}(6.3630+73.175+2.9552)$

= 0.0497

Sd(logit(sp)) = 0.223

Log($\frac{0.8912}{0.1088})$±1.96*0.223= (1.666, 2.5401)

${logit}^{-1}(1.666, 2.5401)$ = (0.8410, 0.9269)

The 95% CI for specificity

**(84.10%, 92.69%)**
