# Supplementary material for: Glycated haemoglobin and fasting plasma glucose tests in the screening of outpatients for diabetes and abnormal glucose regulation in Uganda: A diagnostic accuracy study
Source: PLoS One. 2022 Aug 4;17(8):e0272515. doi: 10.1371/journal.pone.0272515 (PMC9352087; doi:10.1371/journal.pone.0272515)
Supplement: S4 Appendix — (DOCX) [file pone.0272515.s006.docx]

**Supporting File 4: ROC and Interval Likelihood Ratios for the FPG and HbA1c tests when used to screen for Diabetes and AGR**

***Table 1: ROC table for FPG when used to screen for diabetes***

| **Cut offs** | **Sensitivity** | **1-Specificity** |
| --- | --- | --- |
| 0 | 1 | 1 |
| 3 | 1 | 1 |
| 6 | 0.7914 | 0.0421 |
| 6.2 | 0.7914 | 0.0276 |
| 6.4 | 0.7266 | 0.0132 |
| 6.6 | 0.6978 | 0.0099 |
| 6.8 | 0.6547 | 0.0079 |
| 7.0 | 0.6259 | 0.0059 |
| 7.2 | 0.5683 | 0.0033 |
| 7.4 | 0.5252 | 0.0020 |
| 7.6 | 0.5108 | 0.0013 |
| 9 | 0.3957 | 0.0007 |
| 12 | 0.3237 | 0.0007 |
| 15 | 0.1942 | 0.0007 |
| 18 | 0.1223 | 0 |
| 21 | 0.0692 | 0 |
| 24 | 0.0072 | 0 |
| 27 | 0 | 0 |

***Table 2: ROC table for HbA1c when used to screen for AGR***

| **Cut offs** | **Sensitivity** | **1-Specificity** |
| --- | --- | --- |
| 0 | 1 | 1 |
| 15 | 1 | 1 |
| 30 | 0.9928 | 0.8763 |
| 45 | 0.7122 | 0.0487 |
| 46 | 0.7122 | 0.0296 |
| 47 | 0.7050 | 0.0165 |
| 48 | 0.6978 | 0.0145 |
| 49 | 0.6978 | 0.0007 |
| 50 | 0.6475 | 0.0007 |
| 51 | 0.6259 | 0.0007 |
| 60 | 0.4892 | 0 |
| 75 | 0.3525 | 0 |
| 90 | 0.2734 | 0 |
| 105 | 0.0863 | 0 |
| 120 | 0.0611 | 0 |
| 135 | 0 | 0 |

***Table 3: ROC table for FPG when used to screen for AGR***

| **Cut offs** | **Sensitivity** | **1-Specificity** |
| --- | --- | --- |
| 0 | 1 | 1 |
| 3 | 1 | 1 |
| 5.5 | 0.5319 | 0.0731 |
| 5.7 | 0.4681 | 0.0490 |
| 5.9 | 0.3385 | 0.0249 |
| 6 | 0.3385 | 0.0166 |
| 6.1 | 0.2967 | 0.0166 |
| 6.3 | 0.2593 | 0.0091 |
| 6.5 | 0.2440 | 0.0075 |
| 9 | 0.1209 | 0.0008 |
| 12 | 0.0989 | 0.0008 |
| 15 | 0.0593 | 0.0008 |
| 18 | 0.0374 | 0 |
| 21 | 0.0198 | 0 |
| 24 | 0.0021 | 0 |
| 27 | 0 | 0 |

***Table 4: ROC table for HbA1c when used to screen for AGR***

| **Cut offs** | **Sensitivity** | **1-Specificity** |
| --- | --- | --- |
| 0 | 1 | 1 |
| 15 | 1 | 1 |
| 30 | 0.9253 | 0.8713 |
| 42 | 0.4232 | 0.1088 |
| 43 | 0.3487 | 0.0515 |
| 44 | 0.3443 | 0.0349 |
| 45 | 0.3121 | 0.0257 |
| 46 | 0.2895 | 0.010 |
| 47 | 0.2659 | 0.0017 |
| 48 | 0.2593 | 0.0008 |
| 49 | 0.2176 | 0 |
| 60 | 0.1495 | 0 |
| 75 | 0.1077 | 0 |
| 90 | 0.0835 | 0 |
| 105 | 0.0264 | 0 |
| 120 | 0.0180 | 0 |
| 135 | 0 | 0 |

***Table 3: Likelihood Ratio table for diabetes based on the FPG test***

| **Interval** | **#** | **Proportion with diabetes (A)** | **#** | **Proportion without diabetes (B)** | **Interval LR (A/B)** |
| --- | --- | --- | --- | --- | --- |
| ≥27 | 0 | NA | 0 | NA | NA |
| 24-26.9 | 1 | 1 | 0 | 0 | NA |
| 21-23.9 | 8 | 1 | 0 | 0 | NA |
| 18-20.9 | 8 | 1 | 0 | 0 | NA |
| 15-17.9 | 10 | 0.9091 | 1 | 0.0909 | 10.0011 |
| 12-14.9 | 18 | 1 | 0 | 0 | NA |
| 9-11.9 | 10 | 1 | 0 | 0 | NA |
| 6-8.9 | 55 | 0.4661 | 63 | 0.5339 | 0.8730 |
| 3-5.9 | 29 | 0.0195 | 1456 | 0.9805 | 0.0199 |
| 0-2.9 | 0 | NA | 0 | NA | NA |

***Table 4: Likelihood Ratio table for diabetes based on the HbA1c test***

| **Interval** | **#** | **Proportion with diabetes (A)** | **#** | **Proportion without diabetes (B)** | **Interval LR (A/B)** |
| --- | --- | --- | --- | --- | --- |
| ≥135 | 0 | NA | 0 | NA | NA |
| 120-134 | 8 | 1 | 0 | 0 | NA |
| 105-119 | 4 | 1 | 0 | 0 | NA |
| 90-104 | 26 | 1 | 0 | 0 | NA |
| 75-89 | 11 | 1 | 0 | 0 | NA |
| 60-74 | 19 | 1 | 0 | 0 | NA |
| 45-59 | 31 | 0.2952 | 74 | 0.7048 | 0.4188 |
| 30-44 | 39 | 0.0279 | 1258 | 0.9721 | 0.0287 |
| 15-29 | 1 | 0.0053 | 188 | 0.9947 | 0.0053 |
| 0-14 | 0 | NA | 0 | NA | NA |

***Table 5: Likelihood Ratio table for AGR based on the FPG test***

| **Interval** | **#** | **Proportion with diabetes (A)** | **#** | **Proportion without diabetes (B)** | **Interval LR (A/B)** |
| --- | --- | --- | --- | --- | --- |
| ≥27 | 0 | NA | 0 | NA | NA |
| 24-26.9 | 1 | 1 | 0 | 0 | NA |
| 21-23.9 | 8 | 1 | 0 | 0 | NA |
| 18-20.9 | 8 | 1 | 0 | 0 | NA |
| 15-17.9 | 10 | 0.9091 | 1 | 0.0909 | 10.0011 |
| 12-14.9 | 18 | 1 | 0 | 0 | NA |
| 9-11.9 | 10 | 1 | 0 | 0 | NA |
| 6-8.9 | 381 | 0.9525 | 19 | 0.0475 | 20.0526 |
| 3-5.9 | 19 | 0.0158 | 1184 | 0.9842 | 0.0161 |
| 0-2.9 | 0 | NA | 0 | NA | NA |

***Table 6: Likelihood Ratio table for AGR based on the HbA1c test***

| **Interval** | **#** | **Proportion with diabetes (A)** | **#** | **Proportion without diabetes (B)** | **Interval LR (A/B)** |
| --- | --- | --- | --- | --- | --- |
| ≥135 | 0 | NA | 0 | NA | NA |
| 120-134 | 8 | 1 | 0 | 0 | NA |
| 105-119 | 4 | 1 | 0 | 0 | NA |
| 90-104 | 26 | 1 | 0 | 0 | NA |
| 75-89 | 11 | 1 | 0 | 0 | NA |
| 60-74 | 19 | 1 | 0 | 0 | NA |
| 45-59 | 74 | 0.7048 | 31 | 0.2952 | 2.3875 |
| 30-44 | 279 | 0.2151 | 1018 | 0.7849 | 0.2740 |
| 15-29 | 34 | 0.1799 | 155 | 0.8201 | 0.2194 |
| 0-14 | 0 | NA | 0 | NA | NA |
